# Publisher Correction: Variations in Dysbindin-1 are associated with cognitive response to antipsychotic drug treatment

**DOI:** 10.1038/s41467-018-06062-y

**Published:** 2018-08-29

**Authors:** Diego Scheggia, Rosa Mastrogiacomo, Maddalena Mereu, Sara Sannino, Richard E. Straub, Marco Armando, Francesca Managò, Simone Guadagna, Fabrizio Piras, Fengyu Zhang, Joel E. Kleinman, Thomas M. Hyde, Sanne S. Kaalund, Maria Pontillo, Genny Orso, Carlo Caltagirone, Emiliana Borrelli, Maria A. De Luca, Stefano Vicari, Daniel R. Weinberger, Gianfranco Spalletta, Francesco Papaleo

**Affiliations:** 1 Department of Neuroscience and Brain Technologies, Genetics of Cognition laboratory, Istituto Italiano di Tecnologia, via Morego, 30, 16163 Genova, Italy; 2 Dipartimento di Scienze del Farmaco, Universita’ degli Studi di Padova, Largo Meneghetti 2, 35131 Padova, Italy; 3 Lieber Institute for Brain Development, Johns Hopkins University Medical Campus, Baltimore, MD 21205 USA; 4 Department of Neuroscience, Bambino Gesù Children’s Hospital, Piazza Sant’Onofrio 4, 00100 Rome, Italy; 5 IRCCS Santa Lucia Foundation, Neuropsychiatry Laboratory, 00179 Rome, Italy; 6 Research Laboratory for Stereology and Neuroscience, Bispebjerg University Hospital, 2400 Copenhagen, NV Denmark; 7 IRCCS E. Medea Scientific Institute, 23842 Bosisio Parini, Italy; 8 University of California, Irvine, CA 92697 USA; 9 Department of Biomedical Sciences, Università di Cagliari, 09124 Cagliari, Italy; 10 Departments of Psychiatry, Neurology, Neuroscience and the McKusick-Nathans Institute of Genetic Medicine, Johns Hopkins School of Medicine, Baltimore, MD 21205 USA; 11 Menninger Department of Psychiatry and Behavioral Sciences, Baylor College of Medicine, Houston, TX 77030 USA; 12 Center for Psychiatric Neuroscience, Department of Psychiatry, University Hospital Center Lausanne, CH-, 1008 Prilly-Lausanne, Switzerland

Correction to: *Nature Communications* 10.1038/s41467-018-04711-w, published online 11 Jun 2018.

In the original version of this Article, references in the Methods section incorrectly referred to references in the Supplementary References section. The relevant references (now numbered 20, 27, 42, 47, 69–80) have been removed from the Supplementary References section of the Supplementary Information file and added to the References section of the main manuscript, in both the PDF and HTML versions of the Article.

