# Supplementary material for: Variations in Dysbindin-1 are associated with cognitive response to antipsychotic drug treatment
Source: Nat Commun. 2018 Jun 11;9:2265. doi: 10.1038/s41467-018-04711-w (PMC5995960; doi:10.1038/s41467-018-04711-w)
Supplement: Supplementary file 1 — Supplementary Information [file 41467_2018_4711_MOESM1_ESM.pdf]

Variations in Dysbindin-1 are associated with cognitive response to antipsychotic drug treatment

Scheggia et al.

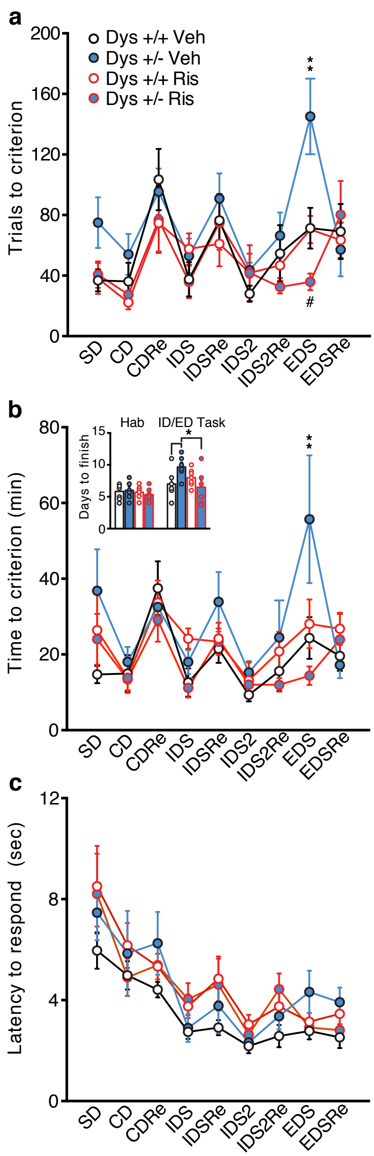

Supplementary Figure 1. Attentional set-shifting after chronic treatment with Veh or Ris in Dys+/+ and +/- mice. **a** Total number of trials, **b** time (in minutes), and **c** latency to respond (in seconds) needed to reach the criteria during the different stages of the ID/ED task grouped by Dys+/+, +/- Veh, and Dys +/+, +/- Ris (n=6-7 each group). Inset, day to complete the habituation (hab) and the ID/ED task. One Dys+/+ Veh, two Dys+/+ Ris, four Dys+/- Veh and four +/- Ris mice were excluded because they failed to complete the entire procedure of the ID/ED task. Two-way RM ANOVA analysis of mice performance in the ID/ED task after chronic Veh or Ris revealed a significant treatment effect for the trials ($F_{(1,21)}=10.05$, $p<0.005$) and time ($F_{(1,21)}=4.44$, $p<0.05$) needed to reach the criterion and for the latency to respond ($F_{(1,46)}=4.10$, $p<0.05$). Moreover, two-way ANOVA analysis of EDS performance showed a significant genotype × treatment effect for the number trials ($F_{(1,21)}=9.05$, $p<0.05$) and for the time ($F_{(1,21)}=3.88$; $p=0.05$) needed to finish the stage. Analysis of the latency to respond during the EDS showed a trend towards a significant treatment × genotype effect (two-way ANOVA: $F_{(1,44)}=3.09$, $p=0.08$). Risperidone treatment had no effect on the performance of Dys+/+ mice in any stage of the task ($p=0.96$). Error bars represent S.E.M. (**a, b**) **$p<0.005$ vs all the other groups, #$p<0.05$ vs Dys+/+ Ris at same stage, *$p<0.05$ vs Dys +/- Veh.

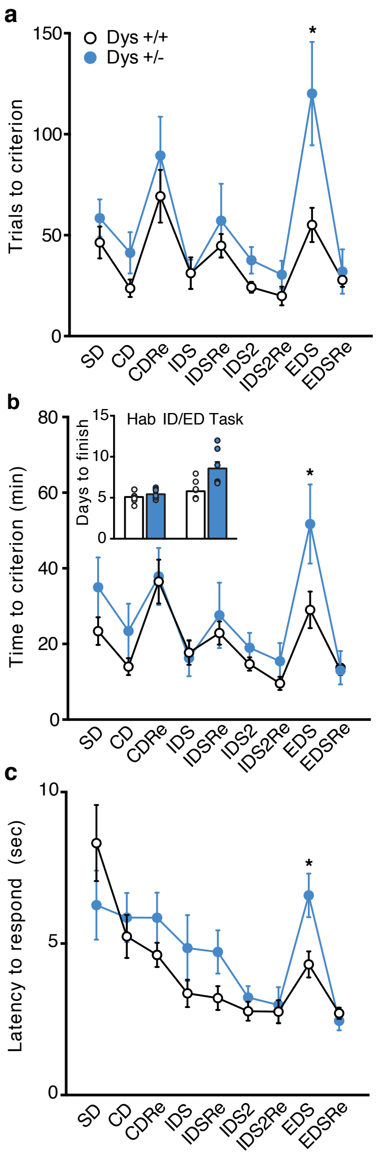

Supplementary Figure 2. Genetic reduction of dysbindin-1 produces selective PFC-dependent attentional set-shifting impairments in mice. **a** Total number of trials, **b** time (in minutes), and **c** latency to respond (in seconds) needed reach the criteria during the different stages of the ID/ED task grouped in Dys+/+ (n=10) and Dys+/- (n=7) mice. Inset, day to complete the habituation (hab) and the ID/ED task. Five Dys+/- mice were excluded because were not able to finish the task, in contrast, only 1 Dys+/+ failed to complete the entire procedure. All the other mice readily learned to poke in the holes for food rewards and were able to perform the multiple sequences of discriminations. Analysis of Dys+/+ performance revealed a significant effect of discrimination (two-way RM ANOVA, trials: $F_{(8,72)}=6.74$, $p<0.0005$; time: $F_{(16,72)}=6.85$, $p<0.0005$). Analysis of the performance on the EDS showed a significant effect of genotype (one-way ANOVA, trials: $F_{(1,15)}=7.69$, $p<0.05$; time: $F_{(1,15)}=4.75$, $p<0.05$). Analysis of the latency to respond showed a significant effect of discrimination ($F_{(8,240)}=11.40$, $p<0.0005$). In particular, as previously reported[3] mice improved their speed to respond over consecutive stages as demonstrated by a significant decreased in latency to poke at the IDS2 compared with initial discrimination (SD). Moreover, analysis of the latency on the EDS revealed a significant effect of genotype (one-way ANOVA, $F_{(1,30)}=8.43$, $p<0.05$). Error bars represent S.E.M. *p <0.05 vs Dys+/+ at same stage.

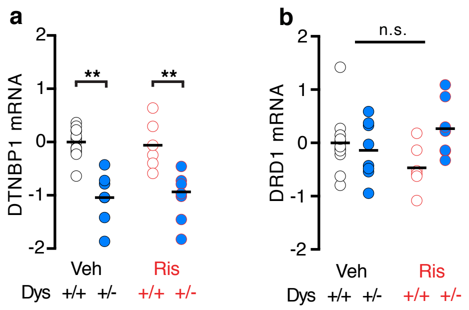

Supplementary Figure 3. *DNTBP1* and *DRD1* gene expression. **a** Relative gene expression of dysbindin-1 gene (*DNTBP1*) in the mPFC of Dys+/+ and +/- mice after chronic Risperidone or Vehicle treatment (n=6-7 each group). Risperidone did not affect dysbindin-1 gene expression in either Dys+/+ or in +/- mice (two-way ANOVA, treatment $F_{(1,28)}$=0.07, p=0.77; genotype treatment $F_{(1,28)}$=30.20, p<0.0005). **p<0.005 vs. Dys+/+. **b** Relative gene expression of dopamine receptor D1 (*DRD1*) in the mPFC of Dys+/+ and +/- mice after chronic Risperidone or Vehicle treatment (n=6-7 each group). Chronic treatment with risperidone did not altered the relative mRNA expression of total D1 receptors (two-way ANOVA, treatment $F_{(1,29)}$=0.00; p=0.97; genotype $F_{(1,29)}$=1.63, p=0.21). Error bars represent S.E.M.

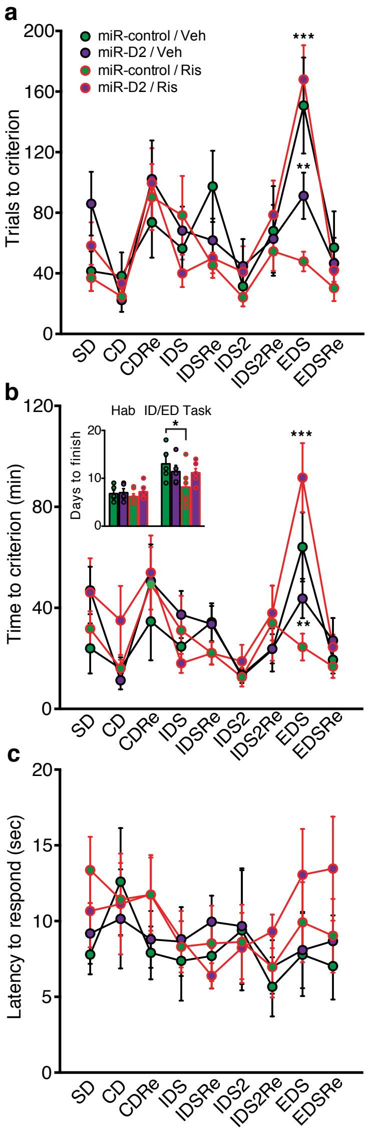

Supplementary Figure 4. Silencing of D2 receptors in the mPFC of Dys+/- mice blocked the beneficial effect of risperidone on attentional set-shifting abilities. **a** Total number of trials, **b** time (in minutes), and, **c** latency to respond (in seconds) needed to reach the criteria during the different stages of the ID/ED task in Dys+/- mice injected with miR-control or miR-D2 after treatment with chronic Veh or Ris (n=5-7 each group). Inset, number of days to finish the Habituation phase and the ID/ED Task. Three miR-control Ris, two miR-D2 Ris and two miR-D2 Veh mice were excluded because they failed to complete the entire procedure of the ID/ED task. Two-way ANOVA analysis of mice performance in the ID/ED task after chronic Veh or Ris revealed a significant group x stage effect for the trials ($F_{(16,120)}$=2.21, $p<0.05$) and time ($F_{(16,120)}$=2.07, $p<0.05$) needed to reach the criterion. No significant effect for the latency to respond ($F_{(16,128)}$=0.89, p=0.57). Error bars represent S.E.M. ***$p<0.0005$ vs miR-control/Ris, **$p<0.005$ vs. miR-D2/Ris).

	Healthy controls subjects			
	Dys Hap+/+	Dys Hap+/-	Dys Hap-/-	
**N**	83	151	80	
**Gender**	46M/37F	69M/82F	41M/39F	
**Age**	43.76 (1.65)	40.63 (1.29)	41.65 (1.64)	$F_{(2,310)}$ =1.11 p=0.32
**Education (Years)**	14.43 (0.42)	14.58 (0.28)	14.94 (0.39)	$F_{(2,310)}$ =0.44 p=0.64
**MMSE score**	29.48(0.09)	29.42 (0.07)	29.56 (0.08)	$F_{(2,310)}$ =0.70 p=0.49

	Patients with Schizophrenia			
	Dys Hap+/+	Dys Hap+/-	Dys Hap-/-	
**N**	76	128	55	
**Gender**	38M/31F	91M/37F	35M/20F	
**Age**	38.76 (1.44)	36.24 (1.14)	38.24 (1.67)	$F_{(2,256)}$ =1.08 p=0.33
**Education (Years)**	11.45 (0.41)	12.30 (0.26)	11.64 (0.37)	$F_{(2,256)}$ =2.04 p=0.13
**MMSE score**	26.85 (0.45)	27.50 (0.31)	27.86 (0.30)	$F_{(2,237)}$ =1.49 p=0.22
**Antipsychotic treatments (CPZ equivalents)**	391.55 (44.61)	572.48 (71.96)	350.84 (44.50)	$F_{(2,224)}$ =0.55 p=0.57
**Treatment duration (years)**	14.43 (1.71)	13.90 (1.29)	13.37 (1.77)	$F_{(2,169)}$ =0.09 p=0.91
**PANSS Pos**	22.74 (0.85)	21.94 (0.66)	22.46 (0.92)	$F_{(2,240)}$ =0.30 p=0.73
**PANSS Neg**	22.82 (0.96)	22.50 (0.69)	21.50 (1.25)	$F_{(2,240)}$ =0.42 p=0.65
**PANSS PG**	49.81 (1.63)	47.90 (1.15)	47.26 (1.61)	$F_{(2,240)}$ =0.73 p=0.48

	First psychotic episode subjects			
	Dys Hap+/+	Dys Hap+/-	Dys Hap-/-	
**N**	15	23	7	
**Gender**	4M/11F	8M/15F	5M/2F	
**Age (Years)**	14.87 (0.65)	15.13 (0.67)	15.43 (0.72)	$F_{(2,41)}$ =0.09 p=0.91
**Education (Years)**	8.87 (0.70)	9.48 (0.63)	9.57 (0.75)	$F_{(2,41)}$ =0.24 p=0.76
**Antipsychotic treatments (equivalents of Ris)**	2.8 (0.7)	2.9 (0.3)	3.2 (0.6)	$F_{(2,42)}$ =0.17 p=0.84
**Treatment duration (weeks)**	3-4	3-4	3-4	

Supplementary Table 1. Summary of the characteristics of the human subjects. Demographic information of healthy volunteers, patients with schizophrenia and patients with first-episode psychosis, genotyped for Dys Hap. Means ± S.E.M

Marker	Allele	Healthy controls subjects Frequency	Healthy controls subjects HWE p value	Patients with Schizophrenia Frequency	Patients with Schizophrenia HWE p value
**rs2619538**	T	0.53	0.9	0.47	0.4
**rs3213207**	A	0.94	0.4	0.92	0.8
**rs1047631**	A	0.89	0.1	0.89	0.8
**Dys Haplotype**	T-A-A (-)	0.50		0.46	
	A-A-A	0.32		0.39	
**Combined**	(A-G-A, A-G-G, T-G-G, T-G-A)	0.18		0.15	
**Dys Diplotype**	T-A-A / T-A-A (-/-)	0.25		0.22	

Supplementary Table 2. Alleles and dysbindin-1 haplotype frequencies. Allele frequencies and p value for the Hardy-Weinberg equilibrium (HWE) for each SNP; and haplotype frequencies in healthy controls subjects and patients with schizophrenia.

	Dys Haplotype non-carriers (+/+) WCST score				Dys Haplotype carriers (+/-,-/-) WCST score			
**Medication**	**0**	**2**	**6**	**18**	**0**	**2**	**6**	**18**
**Risperidone**	-0,011 (0,117)	0,248 (0,121)	0,252 (0,123)	0,350 (0,130)	0,210 (0,234)	0,175 (0,283)	0,386 (0,258)	0,358 (0,354)
**Quetiapine**	0,069 (0,109)	0,027 (0,128)	0,282 (0,124)	0,170 (0,120)	0,952 (0,078)	0,790 (0,168)	0,929 (0,202)	0,978 (0,189)
**Perphenazine**	0,163 (0,124)	0,333 (0,155)	0,301 (0,169)	0,410 (0,170)	0,014 (0,166)	0,310 (0,190)	0,321 (0,208)	0,723 (0,132)
**Olanzapine**	0,142 (0,112)	0,305 (0,107)	0,202 (0,117)	0,390 (0,100)	-0,323 (0,241)	0,046 (0,177)	0,556 (0,235)	0,604 (0,227)

Supplementary Table 3. Cognitive performance of patients enrolled in the CATIE trial. WCST scores in 359 patients recruited into the CATIE trial, grouped by treatment and Dys Hap genotype. We excluded patients assigned to ziprasidone testing because we did not have enough patients with the Dys Hap (n=3) at all the time points of the study

Gene	Dys +/+ Veh	Dys +/- Veh	Dys +/+ Ris	Dys +/- Ris	Genotype	Treatment	Interaction
SLC6A4	1,212 (0,26)	0,680 (0,15)	1,166 (0,49)	1,880 (0,59)	$F_{(1,27)}$ =3.20 p=0.08	$F_{(1,27)}$ =2.74 p=0.10	$F_{(1,29)}$ =0.06 p=0.79
GRIN1	1,019 (0,05)	0,991 (0,15)	0,946 (0,13)	0,993 (0,12)	$F_{(1,29)}$ =0.00 p=0.93	$F_{(1,29)}$ =0.10 p=0.75	$F_{(1,29)}$ =0.11 p=0.73
GRIN2A	1,023 (0,06)	1,045 (0,17)	0,948 (0,14)	1,024 (0,12)	$F_{(1,29)}$ =0.17 p=0.68	$F_{(1,29)}$ =0.16 p=0.68	$F_{(1,29)}$ =0.05 p=0.82
GRIN2B	1,022 (0,06)	1,102 (0,15)	1,045 (0,17)	1,004 (0,09)	$F_{(1,29)}$ =0.02 p=0.87	$F_{(1,29)}$ =0.10 p=0.74	$F_{(1,29)}$ =0.27 p=0.60
HTR2A	1,042 (0,08)	1,050 (0,18)	0,667 (0,05)	1,028 (0,18)	$F_{(1,29)}$ =1.17 p=0.28	$F_{(1,29)}$ =1.40 p=0.24	$F_{(1,29)}$ =2.23 p=0.14

Supplementary Table 4. Relative gene expression, in the mPFC of Dys+/+ and +/- mice after chronic Risperidone or Vehicle treatment (n=6-7 each group) of: serotonin transporter (*SLC6A4*), glutamate ionotropic receptor NMDA type subunit 1 (*GRIN1*), glutamate Ionotropic receptor NMDA type subunit 2A (*GRIN2A*), glutamate ionotropic receptor NMDA type subunit 2B (*GRIN2B*), serotonin receptor 2A (*HTR2A*). Means ± sem, two-way ANOVA (genotype X treatment)
